# The Food Environment and Student Weight Status, Los Angeles County, 2008-2009

**Published:** 2012-02-23

**Authors:** Brent A. Langellier

**Affiliations:** Department of Community Health Sciences, University of California, Los Angeles, School of Public Health

## Abstract

**Introduction:**

One factor believed to affect overweight status is the food environment, or the distribution of outlets that serve healthful or unhealthful foods in residential areas, workplaces, and schools. Few studies have investigated the association between the food environment and the prevalence of overweight among children and adolescents. The objective of this study was to investigate the association between the distribution of corner stores and fast food restaurants around Los Angeles County public schools and the prevalence of overweight among students.

**Methods:**

Hierarchical linear models were used to assess the association between the presence of corner stores or fast food restaurants within a half-mile of Los Angeles County schools (N = 1,694) and overweight prevalence among students in grades 5, 7, and 9.

**Results:**

The presence of corner stores and fast food restaurants varied significantly by schools' racial/ethnic composition, Title 1 eligibility, and rural/suburban vs urban location. After adjustment for other factors, overweight prevalence was 1.6 percentage points higher at majority-Latino schools that had at least 1 corner store within a half-mile than at majority-Latino schools that did not have a corner store within a half-mile. The association between corner stores and overweight prevalence varied significantly between majority-Latino schools and schools that were majority-white or that had no racial/ethnic majority. The presence of fast food restaurants within a half-mile of schools was not associated with overweight prevalence among students.

**Conclusion:**

This study underscores the importance of interventions that seek to improve the healthfulness of corner store inventories and of student purchases.

## Introduction

Overweight and obesity is a leading public health issue in the United States, affecting adults, children, and adolescents (1-3). Recent estimates indicate that 17% of children and adolescents are obese and 32% are overweight or obese (4). One factor believed to affect overweight status is the food environment, or the distribution of outlets that serve healthful or unhealthful foods in residential areas, workplaces, and schools. Previous studies of the association between the food environment and overweight prevalence among adults have had mixed results. Some studies found no significant associations between food outlets and adult overweight (5-9), whereas others found a high prevalence of overweight among adults living in neighborhoods without supermarkets (6-8,10) or with high concentrations of fast food restaurants (6,8,11-14) or small grocery and convenience stores (6,8,15,16).

Despite the abundance of research on adults, few studies have investigated the association between the food environment and the prevalence of overweight among children and adolescents (9,13,14,16). Most studies among children and adolescents have concentrated on the effects of fast food restaurants near the home (9) or school (13,14). No study has considered whether the distribution of corner stores near schools is associated with the prevalence of overweight among the children and adolescents attending those schools. Research has shown, however, that corner stores and other food outlets that serve unhealthful foods tend to cluster near schools (17-21). Research in Los Angeles and other areas has also shown that most corner stores stock limited healthful food options and an ample variety of unhealthful food options (22-24) and that children and adolescents shopping at corner stores typically purchase unhealthful foods (25).

The objective of this study was to investigate the distribution of fast food restaurants and corner stores around Los Angeles (LA) County schools and the association with student overweight. My hypothesis was that students attending schools near corner stores and fast food restaurants have greater access to unhealthful foods and may be at greater risk of overweight than students attending schools that are not near such food outlets.

## Methods

### Data sources

The location of all food outlets in LA County in 2009 was purchased from the Dun & Bradstreet commercial information service by the California Department of Public Health (CDPH) and is publicly available on the CDPH website (26). Businesses were classified as fast food restaurants and corner stores on the basis of retail categories supplied by Dun & Bradstreet. The "fast food restaurants" category included chain and nonchain fast food restaurants, chain and nonchain pizza restaurants, and chain sandwich restaurants and delicatessens. The "corner stores" category included chain and nonchain convenience stores, nonsupermarket grocery stores, and liquor stores. Liquor stores were included in this category because most liquor stores in LA County sell snack foods and nonalcoholic beverages, and most convenience stores and small grocery stores sell alcohol, resulting in few practical differences between the categories. ArcGIS 9.3 (Esri, Redlands, California) was used to geocode the address of each food outlet and all schools in LA County. ArcGIS was also used to create a half-mile road network buffer around each school and determine whether each buffer contained at least 1 fast food restaurant or corner store.

Data on the prevalence of overweight within each school in LA County were obtained from the California Department of Education (CDE) Physical Fitness Testing program from the 2008-2009 school year (27). CDE requires that the Physical Fitness Testing program be administered annually during the months of February through May to all students in California in grades 5, 7, and 9. The purpose of the program is to assess student fitness on the basis of body mass index (BMI), skinfold measurement, and various physical activity tests such as a 1-mile run and push-ups. Student fitness is determined by comparing student performance on each aspect of the test to sex- and age-specific cutoffs established to represent minimum levels of fitness (27). Students were classified as overweight if they had a BMI higher than the sex- and age-specific cutoffs defined by the Physical Fitness Testing program (27).

Demographic data from the Common Core of Data of the National Center for Education Statistics for each school during the 2008-2009 school year were used as control variables in multivariate analyses (28). As a measure of school-level racial/ethnic composition, dummy-coded variables were used to indicate whether each school was racially mixed or whether more than half of the students were white, Latino, black, or Asian. Because the prevalence of overweight varies significantly by race/ethnicity (4), multivariate analysis was used also to control for the percentage of students in each school who were white, black, and Latino. Further controls included whether the school was located in an urban or a rural/suburban neighborhood and whether each school was a high school, middle school, elementary school, or other type of school. Schools were classified as urban if they were inside both an urbanized area and a principal city, as defined by the US Census Bureau. Schools were classified as "other" if they included students in a wider range of grades than a traditional elementary school (kindergarten to 5th grade), middle school (6th to 8th), or high school (9th to 12th). Most "other" schools in the sample included students in kindergarten through 12th grade. As a proxy for school-level socioeconomic status, variables were also included to indicate whether the school was eligible for federal Title 1 program funding and the percentage of students who were eligible for free lunch (income eligibility <130% of federal poverty level) through the National School Lunch Program. As a proxy for neighborhood-level socioeconomic status, analyses controlled for the median annual family income of each school's census tract on the basis of data from the 2005-2009 American Community Survey (29).

Data from students in grades 5, 7, and 9 at 1,694 of the 1,710 public schools in LA County that were included in both the Physical Fitness Testing program data file and the National Center for Education Statistics data file were used. Sixteen (1%) schools were excluded from analyses because they were missing data on Title 1 eligibility status, the percentage of students eligible for the free lunch program, or the median annual family income of the census tract.

### Statistical analyses

All analyses were conducted using Stata version 11.0 (Stata Corp LP, College Station, Texas). Medians and interquartile ranges were calculated for continuous variables and frequencies were calculated for categorical variables. Cross-tabulation was used to assess whether the presence of corner stores and fast food restaurants within a half-mile of schools was associated with school characteristics. The Pearson χ^2^ test was used to evaluate associations between food outlet presence and racial/ethnic majority, type of school, Title 1 eligibility, and school location (urban vs suburban/rural).

Multilevel linear regression models were used to predict the association between fast food restaurants or corner stores and the prevalence of student overweight after controlling for relevant covariates. Schools were nested within LA County health districts to account for observable and unobservable heterogeneity between different areas in the county. The health districts are used by the LA County Department of Public Health to divide the county into 26 areas that vary by factors such as population density and the social characteristics of residents. Multilevel linear regression models included fixed effects at the school level, a random intercept at the health district level, and a random slope at the health district level, allowing for variation in the association between food outlets and overweight prevalence. Among the fixed effects was a set of interaction terms that allowed for the association between food outlets and overweight prevalence to vary by racial/ethnic majority of students at the school. Covariates were chosen on the basis of model fit statistics, including Bayesian information criterion (BIC) and adjusted *R* ^2^, and previous research suggesting that race/ethnicity (4), income (30), age (1,4), and rural residence (31) are associated with overweight status. The appropriateness of the multilevel model was assessed on the basis of a likelihood ratio test comparing the multilevel model to a multivariate linear regression. The likelihood ratio test indicated that the multilevel model was preferable for both fast food restaurants (χ^2^ = 8.7, *P* = .01) and corner store (χ^2^ = 12.0, *P* = .003).

## Results

Among the 26 LA County health districts, disparities existed in the density of populations, corner stores, and fast food restaurants (Figure). In general, areas with the highest population density also tended to have the highest density of food outlets. The median prevalence of overweight among students at LA County schools was 35.2% (Table 1). Nearly 7 in 10 schools had a corner store within a half-mile, and 64% had a fast food restaurant within a half-mile. Approximately 65% of schools were majority-Latino, whereas 11% were majority-white, 3% were majority-black, and 5% were majority-Asian. Most of the schools in the sample were elementary schools (69%). Schools were evenly split between urban and rural/suburban areas. Most schools in the sample (76%) were eligible for the federal Title 1 program, and a median of 59% of students within each school were eligible for the free lunch program. Median household family income was $60,900.

**Figure.**
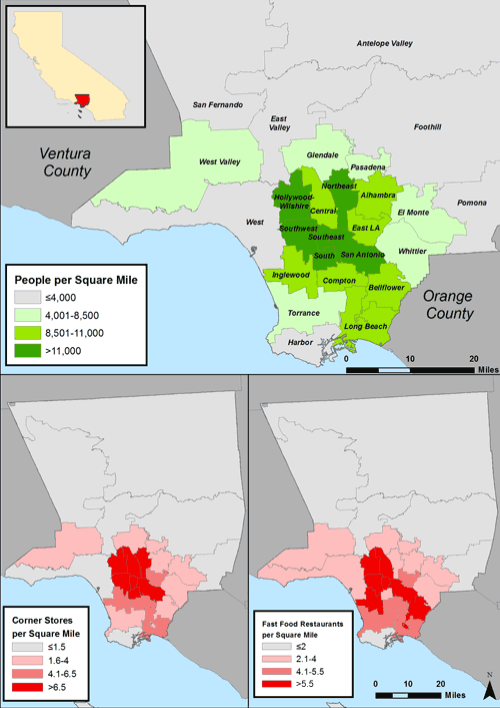
Density of population, corner stores, and fast food restaurants in the 26 Los Angeles County health districts. Data on food outlets are from Dun & Bradstreet (26). Population data are from the US Census Bureau (http://factfinder2.census.gov).

**Table:** 

**Health District**	**Population per Square Mile**	**Corner Stores per Square Mile**	**Fast Food Restaurants per Square Mile**
Compton	10,484.8	5.5	4.5
East LA	8,561.4	6.2	4.9
East Valley	2,187.2	1.1	1.1
El Monte	6,994.1	3.6	3.2
Foothill	631.9	0.2	0.4
Glendale	5,630.1	2.8	3.9
Alhambra	8,604.3	3.3	3.9
Harbor	1,117.6	0.5	0.5
Hollywood-Wilshire	14,718.9	9.7	8.8
Inglewood	10,701.7	5.6	5.9
Long Beach	8,937.0	5.0	5.2
Northeast	11,359.2	6.6	4.0
Antelope Valley	200.0	0.1	0.1
Pasadena	5,634.2	2.1	3.7
Pomona	3,988.2	1.3	2.0
San Antonio	11,725.0	7.0	5.8
Bellflower	8,866.8	3.9	6.0
San Fernando	755.2	0.3	0.5
South	15,278.1	10.8	4.0
Southeast	18,791.7	19.0	5.6
Southwest	13,722.0	8.5	5.8
Torrance	6,453.6	2.9	4.8
West	3,228.9	1.4	2.1
West Valley	4,074.9	1.7	2.7
Central	10934.8	10.2	8.2
Whittier	5,164.3	2.3	3.0

The presence of corner stores and fast food restaurants within a half-mile varied significantly by racial/ethnic majority, Title 1 eligibility, and urban vs rural/suburban location (Table 2). Corner stores were most common among majority-Latino schools (77%), schools eligible for Title 1 funding (75%), and schools in urban areas (77%). Similarly, 69% of majority-Latino schools, 68% of schools eligible for Title 1 funding, and 72% of urban schools were within a half-mile of a fast food restaurant. Corner stores and fast food restaurants were least common among majority-white schools, less than half of which had either type of food outlet within a half-mile.

Model 1 (Table 3) indicates that, after controlling for student and school characteristics, the prevalence of student overweight was 1.6 percentage points higher at majority-Latino schools with at least 1 corner store within a half-mile than at majority-Latino schools without a corner store within a half-mile. The interaction terms in Model 1 indicate that the relationship between the presence of a corner store and overweight prevalence differed significantly between majority-Latino schools and schools that were majority-white or that had no racial/ethnic majority. This relationship did not differ significantly between majority-Latino schools and schools that were majority-black or majority-Asian.

Model 2 of Table 3 suggests that, after controlling for all other factors, the presence of at least 1 fast food restaurant within a half-mile of a majority-Latino school was not associated with a statistically significant difference in the prevalence of student overweight. Interaction terms in Model 2 suggest that the association between the presence of a fast food restaurant and prevalence of student overweight differs significantly between majority-Latino schools and racially/ethnically mixed schools. I found no significant differences in the association between fast food restaurants and the prevalence of student overweight among majority-Latino schools and schools that are majority-white, majority-black, or majority-Asian.

## Discussion

This study adds to the expanding food environment literature by examining the distribution of fast food restaurants and corner stores around schools in LA County. The finding that food outlets that serve unhealthful foods are most commonly found near majority-Latino schools and schools eligible for the federal Title 1 program is consistent with previous national research suggesting that access to fast food restaurants and other sources of unhealthful food is greatest in low-income and minority neighborhoods (6). One reason low-income and Latino schools in LA County may have more access to outlets that serve unhealthful foods is that these populations tend to live in neighborhoods with high population density. As suggested in the Figure, areas with high population density also tend to have high density of corner stores and fast food restaurants.

This study is also among the first to examine the association between unhealthful food outlets around schools and the prevalence of overweight among students (13,14). The finding that the presence of a corner store within a half-mile of majority-Latino schools is associated with significantly higher prevalence of overweight has implications for students and schools. In LA County alone, 65% of all public schools are majority-Latino, and more than 1 million students attend majority-Latino schools. Although an increase of 1.6 percentage points in the prevalence of student overweight may not seem substantial, it actually is: the median prevalence of overweight at public schools in LA County is 35.2%, so an increase in prevalence of 1.6 percentage points represents 4.5% more overweight students.

Some research has examined how the food environment within schools affects what students eat (32), but less research has assessed how student food choices are affected by the food environment surrounding schools. One study demonstrated that youth in Philadelphia tend to purchase nonnutritious, energy-dense foods at corner stores surrounding their schools (25). I found that the presence of at least 1 corner store within a half-mile of a school is associated with a significantly higher prevalence of overweight among students at majority-Latino schools. One possible explanation for this finding is that these students purchase unhealthy food and beverage items at nearby corner stores, which then increases their risk of becoming overweight. My findings and those of Borradaile (25) underscore the importance of current school- and community-based interventions that seek to improve the healthfulness of food and beverage purchases that youth and others make at local corner stores (33-35).

This study has several strengths, one of which is that the data were from LA County, a large, densely packed, and diverse area. At the time of this study, more than 1.5 million students attended the sample of schools, including students from a range of races, ethnicities, and socioeconomic backgrounds. My results may apply to schools in other urban areas. The food environment surrounding schools was measured using half-mile road-network buffers rather than census tracts or buffers based on aerial distance. School-specific buffers are better than administrative units such as census tracts or zip codes because each buffer is the same size and is centered on each school. Each buffer represents the walkable distance around a school, a construct that can be only poorly approximated by administrative units, which are typically either very large, such as zip codes, or highly variable in size, such as census tracts. Furthermore, schools may be located on or near the border of census tracts and zip codes, making such administrative units a poor representation of the physical environment surrounding schools. Road-network buffers are better than buffers based on aerial distance because they more accurately reflect how people travel in the real world — on roads and sidewalks.

This study has several limitations. First, the unit of analysis was neither student nor school but the population of 5th, 7th, and 9th graders within schools. The findings, therefore, may not be generalizable to individual students or the larger student populations of schools in LA County. Another limitation is that the food outlet data came from a commercial data provider and likely included at least some measurement error. Several authors have investigated measurement error in Dun & Bradstreet and other commercial data sets and found that businesses are sometimes omitted entirely or entered with inaccurate location information (36,37). I was unable to verify the accuracy of the business data or the precision with which I geocoded business addresses to geographic points. The data were cross-sectional, and the multivariate models may be subject to bias from unobserved variables. Specifically, food outlets in LA County are clearly distributed in a nonrandom way, and the finding that the presence of corner stores is associated with student overweight at majority-Latino schools may reflect confounding by some unobserved factors. I attempted to limit confounding by including controls for several factors known to be associated with overweight, including race/ethnicity, socioeconomic status, age, and rural/urban location. I also attempted to limit confounding through hierarchical linear models. Finally, the relationship between the distribution of food outlets around schools and the prevalence of student overweight was clearly mediated by consumption of unhealthful foods, a variable that I did not directly measure.

This study showed that the presence of at least 1 corner store around majority-Latino schools in LA County is associated with higher prevalence of overweight among students. The findings emphasize the importance of interventions that seek to improve the healthfulness of corner store inventories as well as the healthfulness of purchases that students make at corner stores.

## Figures and Tables

**Table 1:** Characteristics of Sample of Schools (N = 1,694) in Los Angeles County, 2008-2009 School Year

**Characteristic**	Median (IQR)^a^	%^a^
**No. of students in each school**	660 (480-975)	—
**Percentage of overweight students in each school** b	35.2 (27.0-41.7)	—
**Fast food restaurant located within a half-mile of school**	—	63.9
**Corner store located within a half-mile of school**	—	68.7
**Racial/ethnic majority of schools **		
Latino	—	64.9
White	—	10.9
Black	—	2.9
Asian	—	4.6
No majority	—	16.8
**School type**		
Elementary	—	68.7
Middle	—	15.4
High	—	13.8
Other	—	2.1
**Rural or suburban school location**	—	49.3
**Racial/ethnic composition of each school, %**		
Latino	67.5 (35.5-87.8)	—
White	5.6 (0.9-24.2)	—
Black	4.1 (1.3-12.5)	—
Asian	5.5 (1.5-12.7)	—
**Schools eligible for federal Title 1 program**	—	76.3
**Percentage of students (in each school) eligible for free lunch through the National School Lunch Program**	59.3 (29.9-77.1)	—
**Annual family income^c^ per census tract, $, thousands**	60.9 (43.7-85.5)	—

Abbreviation: IQR, interquartile range.

a Continuous variables are presented as median (IQR); categorical variables are presented as percentages.

b Students were classified as overweight if they had a body mass index greater than the sex- and age-specific cutoffs defined by the California Department of Education Physical Fitness Testing program (27).

c Based on all families living in the same census tract as the school.

**Table 2.** Cross-Tabulation of Presence of Food Outlets Within a Half-Mile of School and School Characteristics, Los Angeles County (N = 1,694), 2008-2009 School Year

Characteristic	Corner Store			Fast Food Outlet			No. of Schools

	No, %	Yes, %	Pearson χ^2^ (*P*)	No, %	Yes, %	Pearson χ^2^ (*P*)	
**Racial/ethnic majority of school**							
No majority	47.9	52.1	113.1 (<.001)	41.5	58.5	49.2 (<.001)	284
White	51.1	48.9		52.7	47.3		184
Latino	22.7	77.3		31.3	68.7		1,099
Black	36.7	63.3		49.0	51.0		49
Asian	42.3	57.7		37.2	62.8		78
**School type**							
High	32.1	67.9	0.2 (.97)	31.6	68.4	2.5 (.48)	234
Middle	32.2	67.8		36.0	64.0		261
Elementary	31.0	69.0		37.1	62.9		1,163
Other	30.6	69.4		36.1	63.9		36
**School eligible for federal Title 1 program**							
No	51.7	48.3	102.6 (<.001)	48.8	51.2	36.4 (<.001)	402
Yes	24.9	75.1		32.2	67.8		1,292
**Urban or rural/suburban school location**							
Rural/suburban	40.0	60.0	58.2 (<.001)	44.0	56.0	43.7 (<.001)	835
Urban	22.8	77.2		28.5	71.5		859

**Table 3.** Models Predicting the Association Between Having at Least 1 Food Outlet Within a Half-Mile of School Location (N = 1,694) and the Prevalence of Student Overweight in Los Angeles County Schools^a^, 2008-2009 School Year

Characteristic	Model 1: Corner or Liquor Stores		Model 2: Fast Food Restaurants	

	β (SE)	*P *value	β (SE)	*P* value
**Fixed Effects**				
**Corner or liquor store within half-mile**	1.63 (0.61)	.007	—	—
**Fast food restaurant within half-mile**	—	—	0.35 (0.52)	.48
**Racial/ethnic majority of school**				
Latino	1 [Reference]	—	1 [Reference]	—
No majority	1.28 (1.05)	.22	1.02 (1.05)	.33
White	0.81 (1.60)	.61	−0.84 (1.57)	.58
Black	−3.93 (2.37)	.10	−5.33 (2.22)	.02
Asian	−0.74 (1.95)	.70	−0.73 (1.97)	.71
**School type**				
Elementary	1 [Reference]	—	1 [Reference]	—
High	−0.63 (0.58)	.28	−0.62 (0.58)	.28
Middle	−0.78 (0.53)	.14	−0.76 (0.53)	.15
Other	3.17 (1.32)	.02	3.18 (1.32)	.02
**Rural/suburban school location**	−1.02 (0.49)	.04	−0.91 (0.48)	.06
**Racial/ethnic composition of school**				
% Latino	0.17 (0.03)	<.001	0.16 (0.03)	<.001
% White	−0.04 (0.03)	.19	−0.05 (0.03)	.15
% Black	0.19 (0.03)	<.001	0.18 (0.03)	<.001
**School eligible for federal Title 1 program**	1.50 (0.63)	.02	1.35 (0.62)	.03
**Students eligible for free lunch**	0.03 (0.02)	.10	0.03 (0.02)	.04
**Annual family income,^b^ per census tract**	−0.02 (0.01)	.06	−0.02 (0.01)	.03
**Interaction**				
Food outlet*no majority	−3.05 (1.08)	.005	−2.64 (1.06)	.01
Food outlet*white	−2.97 (1.29)	.02	0.06 (1.27)	.97
Food outlet*black	0.50 (2.35)	.83	3.04 (2.24)	.16
Food outlet*Asian	−1.72 (1.88)	.36	−2.26 (1.86)	.22
**Random Effects**				
Corner store slope variance	0.24 (0.84)	—	—	—
Fast food restaurant slope variance	—	—	0.37 (0.82)	—
Health district variance	1.57 (0.84)	—	1.20 (0.75)	—
School variance	57.45 (2.02)	—	57.60 (2.02)	—

Abbreviation: SE, standard error.

a Schools are nested within Los Angeles County health districts.

b Annual family income median family income at the census tract level expressed in thousands of dollars.
